# Prognostic value of lesion-specific and proximal coronary segment pericoronary adipose tissue CT Attenuation in ischemic heart disease with angina pectoris

**DOI:** 10.1038/s41598-025-25445-y

**Published:** 2025-11-24

**Authors:** Lingli Wang, Wenfeng He, Siyu Jiang, Kaixiang Su, Yuqing Tang, Caifeng Pang, Xinyue Chen, Xi Liu, Rui Li

**Affiliations:** 1 Department of Radiology, Affiliated Hospital of North Sichuan Medical College and Sichuan Key Laboratory of Medical Imaging, Nanchong, China; 2 Department of Cardiology, Affiliated Hospital of North Sichuan Medical College, Nanchong, China; 3 Key Laboratory of Carcinogenesis and Translational Research (Ministry of Education), Department of Radiology, Peking University Cancer Hospital & Institute, Beijing, China; 4 CT collaboration, Siemens-healthineers, Chengdu, China

**Keywords:** Ischemic heart disease, Pericoronary adipose tissue, Major adverse cardiovascular events, Vascular inflammation, Cardiovascular diseases, Prognostic markers

## Abstract

This study aimed to evaluate the prognostic value of lesion-specific and proximal 40 mm pericoronary adipose tissue attenuation (PCATa) in predicting major adverse cardiovascular events (MACE) in patients with ischemic heart disease presenting with angina pectoris and to compare their predictive performance. This retrospective study included 213 patients with ≥ 50% coronary artery stenosis who underwent coronary computed tomography angiography and digital subtraction angiography between January 2020 and August 2023. MACE was assessed over a median follow-up period of 15 months. Cox proportional hazards models were used to identify MACE risk factors, and the incremental predictive value of PCATa was evaluated using the C-index and global Chi-square statistics. MACE occurred in 72 patients (33.80%). The proximal 40 mm PCATa (LAD: HR = 1.041, LCX: HR = 1.046, RCA: HR = 1.043; all *P* < 0.01) and lesion-specific PCATa (HR = 1.048, *P* = 0.001) were independent predictors of MACE, and their prognostic performance was comparable. In the secondary endpoint analysis, defined as a composite of all-cause mortality, sudden cardiac death, and acute myocardial infarction, only elevated RCA-PCATa (HR = 1.084, 95% CI: 1.026–1.146, *P* = 0.004) was independently associated with adverse outcomes. Risk stratification models integrating PCATa cutoff values and angina status demonstrated robust predictive ability for MACE. Incorporating PCATa into clinical risk models significantly improved the C-index and global Chi-square values for MACE prediction (all *P* < 0.01). These findings suggest that incorporating PCATa to clinical models significantly improves prognostic accuracy, with comparable performance between proximal and lesion-specific PCATa, while RCA-PCATa may provide superior prognostic value for the most severe cardiovascular outcomes.

**Supplementary Information:**

The online version contains supplementary material available at 10.1038/s41598-025-25445-y.

## Introduction

Ischemic heart disease (IHD), characterized by coronary artery narrowing or obstruction, leads to myocardial ischemia, hypoxia, and potential necrosis. Beyond being the most prevalent cardiovascular disease worldwide, it remains the leading cause of death, placing a substantial burden on global healthcare systems^1,2^. In particular, patients with IHD presenting with angina pectoris typically present with significant coronary artery stenosis or obstruction, which is associated with a poor prognosis and may result in severe adverse outcomes such as myocardial infarction, heart failure, or sudden death. Therefore, effectively monitoring disease progression and assessing prognosis, as well as preventing further deterioration in this high-risk population, represent major challenges in clinical management. Although various clinical and laboratory indicators have been utilized in recent years to assess the prognosis of these patients^3^, their predictive efficacy remains controversial. Studies have demonstrated that vascular inflammation plays a key role in the pathogenesis of IHD and serves as a potential marker for plaque instability and fragility^4,5^. This inflammation can lead to plaque rupture, triggering myocardial infarction, and is closely associated with an increased risk of major adverse cardiac events (MACE)^6–8^. Pericoronary adipose tissue attenuation (PCATa), which is measured by coronary computed tomography angiography (CCTA), has been shown to reflect coronary artery inflammation and is considered a non-invasive inflammatory biomarker. Previous studies have confirmed that elevated PCATa values could predict cardiovascular mortality in patients with coronary artery disease (CAD) and hold significant potential for improving cardiovascular risk stratification^9–11^. However, the debate regarding the optimal method for PCATa evaluation in IHD patients with significant ischemic stenosis continues, specifically regarding whether to emphasize the proximal vessel segment or lesion-specific measurements. Historically, research has focused on PCAT surrounding the proximal 40 mm of the right coronary artery (RCA) to estimate global coronary inflammation^12,13^. However, recent findings suggest that lesion-specific PCATa, representing the mean attenuation of perivascular adipose tissue around the plaque, may offer more precise and targeted prognostic insights. Therefore, this study aimed to compare the prognostic value of lesion-specific PCATa with that of proximal coronary segment PCATa in the three major coronary arteries. Additionally, the incremental and comparative predictive efficacy of these PCAT-based models, when integrated with conventional clinical risk models will also be discussed, ultimately enhancing risk stratification and informing novel treatment strategies.

## Results

### Baseline characteristics

A total of 213 patients with a mean age of 62.85 ± 12.04 years were included in the analysis, of whom 117 (54.93%) were male and 98 (46.01%) presented with unstable angina (UA). During a median follow-up period of 15 months (interquartile range: 13–19 months), 72 patients (33.80%) experienced MACE, corresponding to an annual event rate of 24.95%. Specifically, MACE events included 1 case of sudden cardiac death, 15 cases of heart failure requiring hospitalization, 24 cases of unplanned coronary revascularization, 23 cases of acute myocardial infarction, and 9 cases of recurrent ventricular arrhythmia. Among these, secondary endpoints occurred in 24 patients, comprising 1 sudden cardiac death and 23 acute myocardial infarctions. Compared with non-MACE patients, those who experienced MACE were more often male (65.3% vs. 49.6%, *P* = 0.030), had a higher prevalence of diabetes mellitus (63.5% vs. 42.6%, *P* = 0.006), presented more frequently with UA (73.6% vs. 31.9%, *P* < 0.001), and had a history of smoking and excessive alcohol consumption. Study population characteristics are presented in Table 1 and Supplementary Table 4.

**Table 1:** Baseline characteristics according to the presence of MACE.

	All patients *N* = 213	MACE *N* = 72	NO MACE *N* = 141	*P* value
**Clinical characteristics**				
Age(years)	62.85 ± 12.04	64.56 ± 13.06	61.97 ± 11.43	0.079
Gender(male), n(%)	117(54.93)	47(65.28)	70(49.65)	0.030
BMI(kg/m2)	25.18 ± 2.70	24.93 ± 2.62	25.31 ± 2.74	0.619
Hypertension, n (%)	133(62.44)	48(66.67)	85(60.28)	0.363
Diabetes, n (%)	105(49.30)	45(62.50)	60(42.55)	0.006
History of smoking, n (%)	81(38.03)	36(50.00)	45(31.91)	0.011
History of alcohol consumption, n (%)	35(16.43)	21(29.17)	14(9.93)	0.001
**Lipids**,** mmol/L**				
TG	1.84 ± 1.47	2.11 ± 1.19	1.71 ± 0.77	0.001
TC	4.72 ± 1.26	4.66 ± 1.22	4.75 ± 1.28	0.304
LDL	3.26 ± 1.40	2.89 ± 1.17	3.46 ± 1.46	0.047
HDL	1.17 ± 0.45	1.14 ± 0.37	1.18 ± 0.49	0.089
White cell count, *10^9^/L	6.84 ± 1.65	7.13 ± 1.67	6.68 ± 1.62	0.601
**Medication situation**,** n (%)**				
Antiplatelet	40(18.78)	17(23.61)	23(16.31)	0.197
Statins	42(19.72)	16(22.22)	26(18.44)	0.528
β-blocker	16(7.51)	7(9.72)	9(6.38)	0.305
ACEI/ARB	24(11.27)	10(13.89)	14(9.93)	0.387
**Heart rate**,** bpm**	74.23 ± 11.03	74.71 ± 12.26	73.99 ± 10.38	0.186
**Systolic BP**	133.41 ± 23.96	131.96 ± 23.85	134.15 ± 24.07	0.533
**Diastolic BP**	76.50 ± 10.24	76.11 ± 10.92	76.70 ± 9.91	0.269
**Unstable angina**,** n (%)**	98(46.00)	53(73.61)	45(31.91)	< 0.001
**PCATa**,** HU**				
LAD proximal 40 mm	− 78.74 ± 6.86	− 76.99 ± 7.86	− 79.63 ± 6.12	0.040
LCX proximal 40 mm	− 72.76 ± 6.59	− 70.67 ± 6.51	− 73.83 ± 6.38	0.002
RCA proximal 40 mm	− 78.56 ± 7.54	− 76.12 ± 8.85	− 79.80 ± 6.46	0.005
Lesion-specific PCAT	− 75.45 ± 7.19	− 72.69 ± 7.97	− 76.86 ± 6.33	0.001
**PCAT volume, mm** ^**3**^				
LAD proximal 40 mm	1863.14 ± 463.13	1688.71 ± 465.20	1953.50 ± 436.99	< 0.001
LCX proximal 40 mm	1289.21 ± 447.26	1172.75 ± 459.00	1349.52 ± 430.44	0.003
RCA proximal 40 mm	2186.25 ± 548.98	2124.32 ± 589.04	2218.32 ± 526.37	0.316
**Plaque location**,** n (%)**				
LAD	179(84.04)	65(90.28)	114(80.85)	0.054
LCX	108(50.70)	57(79.17)	51(36.17)	0.001
RCA	145(68.08)	61(84.72)	84(59.57)	0.001
**CAD-RADS category**,** n (%)**				< 0.001
3	116(54.46)	14(19.44)	102(72.34)	
4	77(36.15)	45(62.50)	32(22.70)	
5	20(9.39)	13(18.06)	7(4.96)	

P-values obtained from univariate analysis were used to evaluate the associations among various variables, indicating statistically significant differences. These values reflect the differences between the MACE and non-MACE cohorts.

*MACE* major adverse cardiovascular events, *BMI* body mass index, *TG* triglycerides, *TC* total cholesterol, *LDL* low-density lipoprotein, *HDL* high-density lipoprotein, *β-blocker* beta-blocker, *ACEI* angiotensin-converting enzyme inhibitors, *ARB* angiotensin receptor blockers, *BP* blood pressure, *PCATa* pericoronary adipose tissue attenuation, *LAD* left anterior descending artery, *LCX* left circumflex artery, *RCA* right coronary artery, *CAD-RADS* coronary artery disease reporting and data system.

### CCTA imaging characteristics

The repeatability results of PCATa measurements are summarized in Supplementary Table S1, where both intergroup and intragroup results demonstrate good consistency. Therefore, the initial measurements were used for the final data analysis. Compared to patients without MACE, patients with MACE had higher proximal 40 mm PCATa values in the LAD (− 76.99 ± 7.86 HU vs. −79.63 ± 6.12 HU; *P* = 0.040), LCX (− 70.67 ± 6.51 HU vs. −73.83 ± 6.38 HU; *P* = 0.002), and RCA (− 76.12 ± 8.85 HU vs. −79.80 ± 6.46 HU; *P* = 0.005), respectively, as well as higher lesion-specific PCATa (− 72.69 ± 7.97 HU vs. −76.86 ± 6.33 HU; *P* = 0.001). Additionally, the PCAT volume of the LAD and LCX was lower in patients with MACE (*P* < 0.05). Among the 213 patients, the Coronary Artery Disease-Reporting and Data System (CAD-RADS) categories 4 and 5 were more prevalent in the MACE group (Table 1).

### Prognostic value of PCATa

In the univariate Cox regression analysis, the male sex (*P* = 0.028), diabetes mellitus (*P* = 0.004), smoking (*P* = 0.001), alcohol consumption (*P* = 0.023), and UA (*P* < 0.001) were identified as risk factors for MACE. Additionally, higher proximal 40 mm PCATa of the three major coronary arteries and the lesion-specific PCATa (LAD: *P* = 0.008; LCX: *P* = 0.001; RCA: *P* < 0.001; lesion-specific PCATa: *P* < 0.001), lower PCAT volume of the LAD (*P* < 0.001) and LCX (*P* < 0.006), and CAD-RADS category 4 were significantly associated with MACE. In the multivariate Cox regression analysis, after adjusting for clinical risk factors, proximal 40 mm PCATa of the three major coronary arteries and lesion-specific PCATa remained significantly associated with MACE (LAD: hazard ratio (HR) = 1.041, 95% confidence interval (CI): 1.012–1.071, *P* = 0.006; LCX: HR = 1.046, 95% CI: 1.012–1.082, *P* = 0.008; RCA: HR = 1.043, 95% CI: 1.015–1.073, *P* = 0.003; lesion-specific PCATa: HR = 1.048, 95% CI: 1.018–1.078, *P* = 0.001). Additionally, after adjusting for clinical risk factors, the LAD PCAT volume (HR = 0.999, 95% CI: 0.999–1.000, *P* = 0.010) and LCX PCAT volume(HR = 0.999, 95% CI: 0.999–1.000, *P* = 0.039) were independently associated with MACE (Table 2). For secondary endpoints, elevated RCA-PCATa (HR = 1.084; 95% CI: 1.026–1.146; *P* = 0.004) and reduced LAD PCAT volume (HR = 0.998; 95% CI: 0.997–0.999; *P* = 0.004) were identified as independent predictors (Supplementary Table S2).

**Table 2:** Univariable and multivariable Cox proportional hazard regression analyses for MACE.

	Univariable analysis		Multivariable analysis	
	HR (95% CI)	*P* value	HR (95% CI)	*P* value
Age (per 1 year increase)	1.012(0.992 ~ 1.074)	0.247	–	–
Gender(male)	1.724(1.060 ~ 2.803)	0.031	–	–
BMI (per 1 kg/m2)	0.971(0.890 ~ 1.060)	0.512	–	–
Diabetes	2.010(1.245 ~ 3.244)	0.007	–	–
Hypertension	1.055(0.644 ~ 1.730)	0.831	–	–
History of smoking	1.703(1.072 ~ 2.706)	0.024	–	–
History of alcohol consumption	2.481(1.496 ~ 4.116)	0.023	2.391(1.440 ~ 3.939)	0.001
TG, mmol/L	1.102(0.992 ~ 1.224)	0.071	–	–
Unstable angina	4.132(2.442 ~ 6.992)	< 0.001	4.076(2.407 ~ 6.903)	0.001
PCATa, HU				
LAD proximal 40 mm	1.042(1.011 ~ 1.074)	0.008	1.041(1.012 ~ 1.071)	0.006
LCX proximal 40 mm	1.060(1.025 ~ 1.096)	0.001	1.046(1.012 ~ 1.082)	0.008
RCA proximal 40 mm	1.050(1.022 ~ 1.079)	< 0.001	1.043(1.015 ~ 1.073)	0.003
Lesion-specific	1.062(1.033 ~ 1.093)	< 0.001	1.048(1.018 ~ 1.078)	0.001
PCAT volume, mm3				
LAD proximal 40 mm	0.999(0.999 ~ 1.000)	< 0.001	0.999(0.999 ~ 1.000)	0.010
LCX proximal 40 mm	0.999(0.999 ~ 1.000)	0.006	0.999(0.999 ~ 1.000)	0.039
RCA proximal 40 mm	1.000(0.999 ~ 1.000)	0.135	–	–
CAD-RADS category				
3	1 (reference)	NA	1 (reference)	NA
4	5.822(3.192 ~ 10.622)	< 0.001	5.738(3.144 ~ 10.474)	< 0.001
5	7.334(3.438 ~ 15.646)	< 0.001	5.675(2.582 ~ 12.472)	< 0.001

*BMI* body mass index, *TG* triglycerides, *PCATa* pericoronary adipose tissue attenuation, *LAD* left anterior descending artery, *LCX* left circumflex artery, *RCA* right coronary artery, *CAD-RADS*, coronary artery disease reporting and data system.

**Fig. 1:**
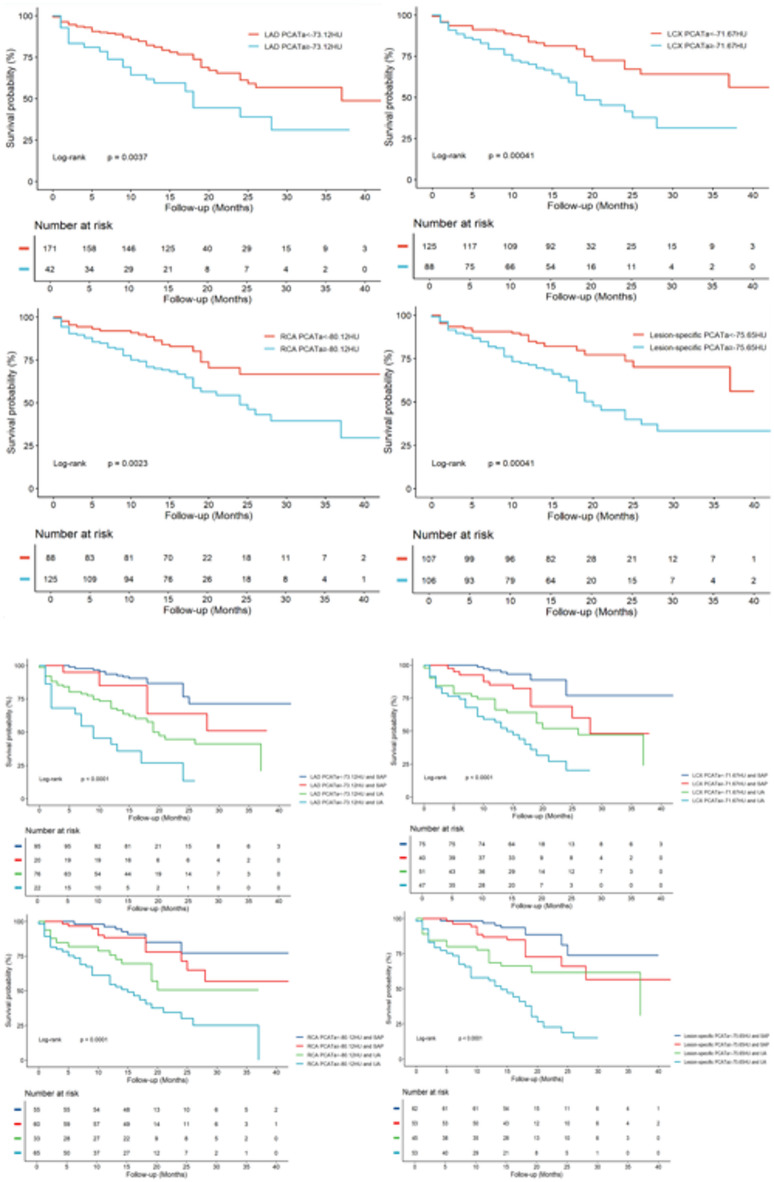
**a** Kaplan-Meier curves illustrating MACE-free survival probability based on PCATa levels. *LAD* left anterior descending artery, *LCX* left circumflex artery, *RCA* right coronary artery. **b** Cumulative survival curves stratified by the presence of UA and PCATa. Stratification was based on PCATa cutoff values: LAD ≥ –73.12 HU or < –73.12 HU with SAP or UA; LCX ≥ –71.67 HU or < –71.67 HU with SAP or UA; RCA ≥ –80.12 HU or < –80.12 HU with SAP or UA; and lesion-specific PCATa ≥ –75.65 HU or < –75.65 HU with SAP or UA. The numbers above the x-axis (in the lower panel) indicate the number of subjects at risk for MACE at a given time. *UA* unstable angina, *SAP* stable angina pectoris, *PCATa* pericoronary adipose tissue attenuation.

The optimum cutoff values for PCATa to predict MACE were determined using the Youden index as follows: −73.12 HU for the LAD proximal 40 mm, − 71.67 HU for the LCX proximal 40 mm, − 80.12 HU for the RCA proximal 40 mm, and − 75.65 HU for lesion-specific PCATa. The Kaplan–Meier curve for the higher PCATa groups showed a significant decrease in the MACE-free survival probability compared with the lower groups (Fig. 1a). After adjusting for clinical risk factors, higher PCATa was associated with an increased risk of MACE (LAD: adjusted HR = 1.425, 95% CI: 1.100–1.844, *P* = 0.009; LCX: adjusted HR = 1.373, 95% CI: 1.080–1.746, *P* = 0.010; RCA: adjusted HR = 1.341, 95% CI: 1.032–1.742, *P* = 0.028; lesion-specific PCATa: adjusted HR = 1.979, 95% CI: 1.184–3.308, *P* = 0.009; Table 3). Because UA is also a significant clinical predictor of MACE, we developed a new predictive model for MACE based on UA and PCATa as follows: Higher PCATa combined with UA was associated with a significantly lower survival rate free from MACE (log-rank test, all *P* < 0.0001). Furthermore, even in the UA group, elevated PCATa was associated with an increased risk of MACE (LAD: HR = 2.206, 95% CI: 1.215–4.006, *P* = 0.009; LCX: HR = 1.807, 95% CI: 1.037–3.147, *P* = 0.037; RCA: HR = 2.021, 95% CI: 1.078–3.791, *P* = 0.028; lesion-specific PCATa: HR = 2.383, 95% CI: 1.318–4.307, *P* = 0.004; Fig. 1b; Table 3).

**Table 3:** Univariate and multivariable analysis for MACE according to the different risk stratification Models.

	Univariable analysis		Multivariable analysis	
	Unadjusted HR (95% CI)	*P* value	Adjusted HR (95% CI)	*P* value
**LAD proximal 40 mm PCATa**				
PCATa < –73.12 HU	1 (reference)	NA	1 (reference)	NA
PCATa ≥ –73.12 HU	1.434(1.116 ~ 1.844)	0.005	1.425(1.100 ~ 1.844)	0.009
PCATa < –73.12 HU and SAP	1 (reference)	NA	1 (reference)	NA
PCATa ≥ –73.12 HU and SAP	1.988(0.754 ~ 5.241)	0.165	–	–
PCATa < –73.12 HU and UA	4.079(2.164 ~ 7.687)	< 0.001	4.092(2.172 ~ 7.711)	< 0.001
PCATa ≥ –73.12 HU and UA	9.477(4.527 ~ 19.837)	< 0.001	8.255(3.907 ~ 17.442)	< 0.001
**LCX proximal 40 mm PCATa**				
PCATa < –71.67 HU	1 (reference)	NA	1 (reference)	NA
PCATa ≥ –71.67 HU	1.503(1.189 ~ 1.901)	0.009	1.373(1.080 ~ 1.746)	0.010
PCATa < –71.67 HU and SAP	1 (reference)	NA	1 (reference)	NA
PCATa ≥ –71.67 HU and SAP	2.760(1.108 ~ 6.873)	0.029	2.863(1.148 ~ 7.138)	0.024
PCATa < –71.67 HU and UA	4.834(2.157 ~ 10.834)	< 0.001	5.142(2.290 ~ 11.542)	< 0.001
PCATa ≥ –71.67 HU and UA	9.148(4.166 ~ 20.085)	< 0.001	8.418(3.817 ~ 18.565)	< 0.001
**RCA proximal 40 mm PCATa**				
PCATa < –80.12 HU	1 (reference)	NA	1 (reference)	NA
PCATa≥ –80.12 HU	1.473(1.138 ~ 1.907)	0.009	1.341(1.032 ~ 1.742)	0.028
PCATa < –80.12 HU and SAP	1 (reference)	NA	1 (reference)	NA
PCATa ≥ –80.12 HU and SAP	1.636(0.644 ~ 4.156)	0.301	–	–
PCATa < –80.12 HU and UA	3.302(1.315 ~ 8.290)	0.011	3.447(1.372 ~ 8.656)	0.008
PCATa ≥ –80.12 HU and UA	7.014(3.134 ~ 15.698)	< 0.001	6.537(2.915 ~ 14.660)	< 0.001
**Lesion-specific PCATa**				
PCATa < –75.65 HU	1 (reference)	NA	1 (reference)	NA
PCATa ≥ –75.65 HU	2.341(1.433 ~ 3.824)	0.001	1.979(1.184 ~ 3.308)	0.009
PCATa < –75.65 HU and SAP	1 (reference)	NA	1 (reference)	NA
PCATa ≥ –75.65 HU and SAP	2.064(0.812 ~ 5.244)	0.128	–	–
PCATa < –75.65 HU and UA	3.723(1.542 ~ 8.986)	0.003	3.979(1.644 ~ 9.629)	0.002
PCATa ≥ –75.65 HU and UA	8.939(3.965 ~ 20.151)	< 0.001	7.867(3.459 ~ 17.889)	< 0.001

*UA* unstable angina, *SAP* stable angina pectoris, *PCATa* pericoronary adipose tissue attenuation, *LAD* left anterior descending Artery, *LCX* left circumflex artery, *RCA* right coronary artery.

To further validate these associations, a sensitivity analysis was conducted by censoring patients who underwent revascularization within 90 days post-CCTA. Notably, both RCA-PCATa and lesion-specific PCATa remained significant independent predictors of MACE (adjusted HR = 1.078 and 1.060, respectively; both *P* < 0.001), while LAD- and LCX-PCATa lost statistical significance (*P* > 0.05; Supplementary Table S3). Corresponding Kaplan–Meier curves confirmed worse MACE-free survival in patients with elevated RCA-PCATa (> − 79.43 HU; *P* = 0.023) and lesion-specific PCATa (> − 80.02 HU; *P* = 0.0076) after exclusion of patients undergoing early revascularization (Supplementary Figure S1a–b).

In addition, survival analysis for the secondary endpoints revealed a significantly worse prognosis in patients with elevated RCA-PCATa (≥ − 80.05 HU) compared to those with lower values (log-rank *P* < 0.0001; Supplementary Figure S2a). When further stratifying patients based on both UA status and RCA-PCATa levels, those with concomitant UA and elevated RCA-PCATa demonstrated the poorest event-free survival, whereas those without UA and with low RCA-PCATa had the most favorable outcomes (log-rank *P* < 0.0001; Supplementary Figure S2b). These findings suggest that RCA-PCATa provides additional prognostic value for hard cardiovascular events, independent of UA status.

### Incremental prognostic value of the proximal 40-mm PCATa and lesion-specific PCATa

**Fig. 2:**
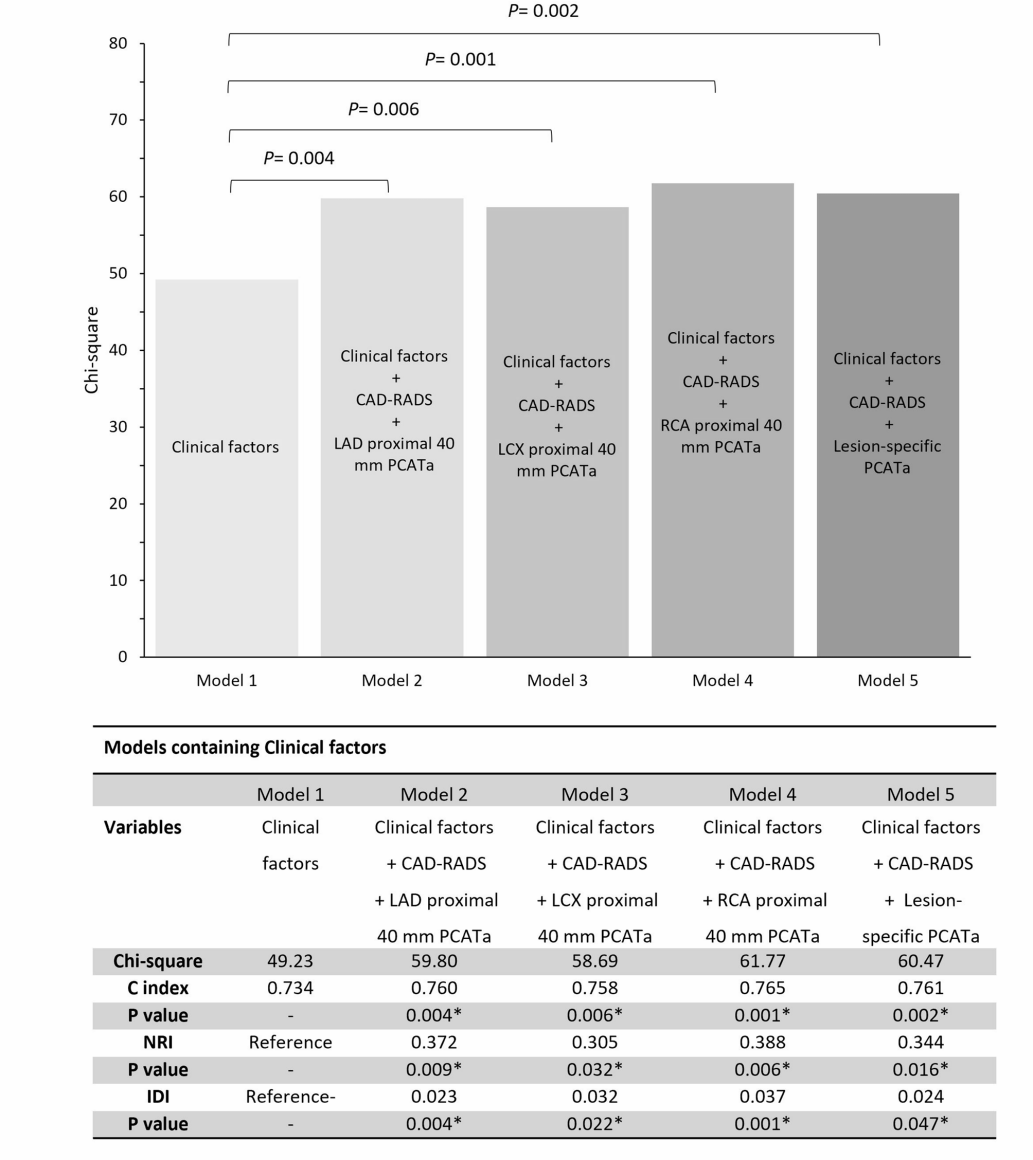
Incremental prognostic value of PCATa values for the proximal 40 mm measurements and lesion-specific measurement. *IDI* integrated discrimination improvement, *NRI* net reclassification index, *CAD-RADS* coronary artery disease reporting and data system, *C index* concordance index.

We developed prognostic models based on clinical risk factors and imaging indicators to predict the risk of MACE in patients presenting with angina pectoris. As shown in Table 4, the addition of the proximal 40 mm PCATa of the three coronary arteries (Models 2, 3, and 4) and lesion-specific PCATa (Model 5) to Model 1 (clinical risk factors) resulted in a significant incremental reclassification benefit (all *P* < 0.05). This integration substantially improved the predictive efficacy of Models 2–5 relative to Model 1 alone, as evidenced by the enhanced performance metrics (Fig. 2). Additionally, comparing the predictive performance of the lesion-specific PCATa model with the proximal 40-mm PCATa model, we found no significant difference in predictive efficacy (all *P* > 0.05).

**Table 4:** Predictive model for MACE based on multivariable Cox proportional hazards regression analysis.

	Model 1		Model 2		Model 3		Model 4		Model 5	
Variable	HR (95%CI)	*P* value	HR (95%CI)	*P* value	HR (95%CI)	*P* value	HR (95%CI)	*P* value	HR (95%CI)	*P* value
Age(per 1 year increase)	1.007(0.987 ~ 1.028)	0.481	1.004(0.984 ~ 1.024)	0.721	1.006(0.985 ~ 1.027)	0.564	1.002(0.982 ~ 1.023)	0.821	1.003(0.983 ~ 1.024)	0.764
Male	1.556(0.786 ~ 3.079)	0.584	1.565(0.595 ~ 3.096)	0.198	1.386(0.703 ~ 2.733)	0.345	1.628 (0.811 ~ 3.269)	0.171	1.489(0.763 ~ 2.906)	0.244
Diabetes	1.265(0.753 ~ 2.123)	0.698	1.008(0.598 ~ 1.699)	0.976	1.048(0.623 ~ 1.763)	0.860	0.966(0.572 ~ 1.631)	0.897	0.984(0.581 ~ 1.666)	0.984
History of smoking	0.903(0.525 ~ 2.074)	0.825	0.837(0.419 ~ 1.674)	0.615	0.833(0.417 ~ 1.662)	0.604	0.762(0.372 ~ 1.560)	0.457	0.841(0.424 ~ 1.670)	0.621
History of alcohol consumption	1.829(1.071 ~ 3.122)	0.027	1.706(1.009 ~ 2.884)	0.010	1.920(1.126 ~ 3.274)	0.017	1.888(1.113 ~ 3.204)	0.018	1.808(1.062 ~ 3.078)	0.029
Unstable angina	4.234(2.500 ~ 7.172)	< 0.001	2.702(1.506 ~ 4.848)	0.001	2.112(1.013 ~ 4.402)	0.046	2.089(1.008 ~ 4.326)	0.047	2.185(1.071 ~ 4.458)	0.032
CAD-RADS category										
3			1 (reference)	NA	1 (reference)	NA	1 (reference)	NA	1 (reference)	NA
4			5.508(3.010 ~ 10.080)	< 0.001	5.203(2.833 ~ 9.556)	< 0.001	5.541(3.033 ~ 10.122)	< 0.001	5.199(2.828 ~ 9.557)	< 0.001
5			5.872(2.673 ~ 12.899)	< 0.001	5.567(2.529 ~ 12.256)	< 0.001	5.889(2.701 ~ 12.844)	< 0.001	5.174(2.347 ~ 11.407)	< 0.001
LAD proximal 40 mm PCATa			1.033(1.003 ~ 1.065)	0.031						
LCX proximal 40 mm PCATa					1.044(1.009 ~ 1.079)	0.012				
RCA proximal 40 mm PCATa							1.047(1.018 ~ 1.077)	0.002		
Lesion-specific PCATa									1.040(1.010 ~ 1.070)	0.008

*LAD* left anterior descending artery, *LCX* left circumflex artery, *RCA* right coronary artery, *CAD-RADS* coronary artery disease reporting and data system, *PCATa* pericoronary adipose tissue attenuation.

## Discussion

In this study, we recruited 213 IHD patients with angina pectoris to investigate the relationship between proximal and lesion-specific PCATa and adverse cardiovascular outcomes. Our findings showed that the proximal 40 mm PCATa of the three coronary arteries and lesion-specific PCATa were independent risk factors for MACE, with equivalent predictive performance. Even among patients with UA, a risk stratification model based on PCATa cutoff values demonstrated improved identification of high-risk IHD patients. Furthermore, an integrated predictive model combining clinical risk factors and PCATa significantly enhanced predictive capability.

In recent years, PCAT has garnered increasing attention due to its pivotal role in cardiovascular risk assessment. As a dynamic endocrine and paracrine organ, PCAT in patients with CAD typically exhibits enhanced inflammatory activity, characterized by the increased release of pro-inflammatory mediators^18^. Histopathological studies have confirmed that vascular inflammation induces a “cachexia-like” phenotype in PCAT, with reduced lipid content and increased aqueous tissue due to adipocyte remodeling^19^. Previous studies have consistently demonstrated that PCAT expresses significantly higher levels of CD31 and MCP-1 compared to other adipose depots, reinforcing its unique pro-inflammatory phenotype in CAD pathogenesis^20^. These inflammatory mediators can infiltrate the adjacent coronary arterial wall, impair endothelial function, and accelerate the progression of atherosclerosis^21^, ultimately contributing to coronary stenosis, myocardial ischemia, and infarction. In addition to its inflammatory role, PCAT is also closely linked to dysregulated lipid metabolism during atherogenesis. It secretes various pro-inflammatory cytokines and fatty acids, such as tumor necrosis factor-α and interleukin-6, which may directly modulate the local immune and metabolic microenvironment of the coronary arteries^22^. These alterations can exacerbate endothelial dysfunction and inflammatory cell infiltration, and may also promote plaque instability through mechanisms such as vascular smooth muscle cell proliferation, collagen degradation, and lipid core expansion. Notably, changes in PCAT inflammatory activity and lipid homeostasis can be visualized noninvasively using imaging techniques, most prominently as increased CT attenuation values^23^. Previous studies have validated PCAT attenuation as an imaging biomarker of vascular inflammation, capable of capturing the dynamic pathobiological processes of atherosclerosis. It complements the anatomical and functional insights provided by CCTA^23^.

Our analysis showed that only RCA-PCATa was independently associated with both MACE and secondary endpoints, highlighting its prognostic value as an imaging surrogate marker of coronary inflammation. Owing to its stable anatomical course and consistent epicardial fat coverage, the proximal RCA is commonly used for PCAT quantification and has been considered a reliable site for assessing coronary inflammation. Inflammatory activity in this region may promote plaque instability and subsequent adverse cardiovascular events^24^. Elevated RCA-PCATa reflects a greater systemic inflammatory burden, particularly in patients prone to life-threatening outcomes, and may even outperform lesion-specific PCATa in predictive utility. These findings are in line with previous studies, such as that by Tzolos et al.^9^, who reported that RCA-PCATa > − 70.5 HU was an independent predictor of myocardial infarction (HR = 2.45, *P*< 0.01). Wen et al^25^. conducted a 3-year follow-up study involving 1,313 patients with acute chest pain, during which 142 patients experienced MACE. After adjusting for clinical risk factors, RCA-PCATa was an independent predictor of MACE (HR = 1.033, *P* = 0.006). We also found that PCAT attenuation in the proximal 40 mm segments of both the LAD and LCX was significantly associated with MACE This result may be attributed to the fact that the proximal segments of all three major coronary arteries are closely surrounded by epicardial adipose tissue, facilitating efficient diffusion of inflammatory mediators through the “adventitial conduit” mechanism^23^. As the initial portion of the coronary arteries, PCAT in these segments may reflect both systemic inflammatory status and local vascular responses^26^. In addition, this observation may be related to the location of culprit plaques, their vulnerability, and subsequent plaque progression^6,7^. This notion is further supported by the findings of Hoshino et al.^11^, who demonstrated that increased LAD-PCATa was a significant predictor of cardiovascular events in a cohort of 220 patients with moderate LAD stenosis. Their results suggest that the proximal vascular PCATa reflects the overall inflammatory activity of the coronary arteries, which may increase plaque vulnerability through mechanisms such as fibrous cap weakening and lipid core content augmentation, thereby promoting atherosclerosis progression and leading to adverse outcomes^27–29^. These findings highlight the role of PCATa in the proximal 40 mm region of the coronary artery as a predictor of coronary inflammation and plaque vulnerability. Moreover, our findings demonstrated that the PCAT volumes within the proximal 40 mm segments of the LAD and LCX were significantly lower in patients with MACE compared to those without, consistent with the results reported by Yu Sun et al^30^.. This phenomenon may be attributed to inflammatory factors derived from the vascular wall that inhibit adipocyte differentiation, resulting in a predominance of immature preadipocytes with smaller size and reduced lipid content in advanced atherosclerosis. In addition, chronic hypoxia and adverse hemodynamic conditions may impair pericoronary adipose tissue homeostasis and accelerate adipocyte apoptosis, thereby further contributing to PCAT volume reduction^30^. Notably, reduced LAD PCAT volume was also associated with an increased risk of secondary endpoints, suggesting that, given the LAD’s critical role in myocardial perfusion, it may serve as a sensitive imaging marker for identifying patients at higher risk of cardiovascular events.

However, although proximal coronary segment PCATa provides an overall assessment of coronary inflammation for disease status and risk stratification, lesion-specific PCATa may offer greater clinical relevance at the individual patient level, particularly for the early identification of plaque rupture risk. Therefore, our study further incorporated lesion-specific PCATa into the analysis and compared its prognostic performance with that of proximal PCATa measurements. The results demonstrated that both proximal and lesion-specific PCATa values across the three major coronary arteries exhibited favorable prognostic value for predicting MACE. Consistent with our findings, Chen et al^16^. also reported that the lesion-specific PCATa value significantly improved the prediction of MACE risk in patients with CAD, outperforming the traditional RCA and LAD proximal 40 mm PCATa values. Although previous studies have demonstrated the prognostic value of lesion-specific PCATa, which may even outperform measurements from proximal coronary segments, our findings indicate that the predictive performance of PCATa is comparable between lesion-specific and proximal assessments. This may be because previous studies primarily focused on patients with suspected CAD (such as those presenting with acute chest pain) who generally have milder conditions with coronary inflammation often concentrated around the lesion plaque. As a result, lesion-specific PCATa values can better reflect localized inflammation, thereby improving prognosticative abilities^31^. In contrast, our study included IHD patients with angina pectoris and coronary artery stenosis in excess of 50%, who had more severe coronary ischemia, larger lesion plaque areas, and higher overall levels of inflammation. Under these conditions, proximal and lesion-specific PCATa demonstrated comparable prognostic value. Given the potential influence of CCTA-guided therapeutic decisions on outcome classification, particularly revascularizations occurring soon after the index test, it was necessary to evaluate whether PCATa retains its prognostic value in a purely observational context. To further validate these observations, we performed a sensitivity analysis excluding patients who underwent early coronary revascularization within 90 days post-CCTA. The results further confirmed the robustness of RCA-PCATa and lesion-specific PCATa as independent predictors of MACE. After censoring early revascularization cases, both RCA-PCATa and lesion-specific PCATa remained robust and statistically significant predictors of MACE, confirming their stability even in medically managed patients. In contrast, the statistical significance of LAD- and LCX-PCATa was attenuated, likely due to the reduced number of events and possible anatomical variability in inflammatory burden. Notably, RCA-PCATa may reflect a broader systemic inflammatory load or high-risk plaque burden^24^, particularly within the RCA territory. In contrast, lesion-specific PCATa directly captures localized inflammation adjacent to culprit lesions, thereby offering enhanced prognostic specificity. These findings underscore the greater clinical utility of RCA and lesion-specific PCATa in risk stratification, especially among patients managed conservatively without early revascularization.

Additionally, we found that the overall predictive performance was significantly improved by combining CCTA imaging markers (CAD-RADS classification and PCATa) with clinical factors. Previous studies have demonstrated that CCTA is important in describing coronary artery anatomy, stenosis severity, and plaque composition^32^. The evaluation based on CCTA and the CAD-RADS classification system has significantly enhanced the non-invasive assessment of CAD; however, these methods primarily focus on structural diseases and fail to fully capture the dynamic changes in the inflammation status^33,34^. Our study further expands on this area by incorporating PCATa values, which improves the predictive model’s effectiveness, suggesting that PCATa can complement traditional anatomical evaluations and enable more accurate risk assessment. Our further analysis revealed that even in patients with UA, higher PCATa values still provided a significant prognostic value. This indicates that PCATa is not only crucial for predicting outcomes in CAD patients but also has strong risk prediction capabilities for high-risk UA patients^26^. As a high-risk phenotype of IHD, patients with UA are prone to acute myocardial infarction and other cardiovascular events due to the instability of coronary artery plaques^26,35^. In this context, PCATa, as a marker reflecting coronary inflammation, can further refine the risk stratification of patients with UA and provide more precise support for clinical decision-making. Future research should further explore the combined use of PCATa with other inflammatory markers and assess its applicability in different populations and types of coronary lesions to fully evaluate its potential value in clinical practice.

Nevertheless, this study has several limitations. First, it is a single-center retrospective analysis conducted in a relatively small Chinese population, which may not represent other centers or racial groups. Second, lesion-specific PCATa was defined as the average attenuation of the perivascular adipose tissue around all plaque vessels, potentially overlooking inherent differences in PCATa among the three major coronary arteries. Third, the study primarily focused on patients with significant coronary artery stenosis, limiting the applicability of its findings to individuals with less severe or asymptomatic disease. Fourth, this study has a relatively low events-per-variable ratio in the multivariate models, which may increase the risk of model overfitting. Although the EPV approached the commonly recommended threshold, the results should be interpreted with caution and require validation in larger, independent cohorts.

## Conclusion

In IHD patients with angina pectoris, the lesion-specific PCATa and proximal 40 mm PCATa in the three coronary arteries were independent predictors of MACE and had comparable prognostic value. Only RCA-PCATa independently predicted secondary endpoints, underscoring its value for identifying patients at risk of severe cardiovascular outcomes. Adding PCATa measures to clinical risk factor models markedly enhances the predictive accuracy.

## Methods

### Study design and patient population

**Fig. 3:**
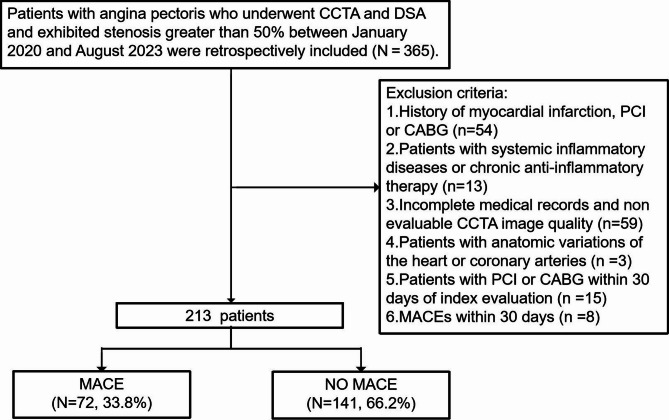
Flow chart showing inclusion and exclusion criteria for the study population. *CCTA* coronary computed tomography angiography, *DSA* digital subtraction angiography, *PCI* percutaneous coronary intervention, *CABG* coronary artery bypass grafting, *MACE* major adverse cardiovascular events.

Patients with angina pectoris who underwent CCTA and digital subtraction angiography (DSA) between January 2020 and August 2023 were retrospectively included. Inclusion Criteria: (1) Patients who underwent both coronary artery DSA and CCTA, with at least one lesion in the three major coronary arteries (diameter ≥ 2 mm) exhibiting stenosis > 50%. (2) Patients presenting with symptoms of myocardial ischemia (e.g., chest pain, dyspnea, or syncope), with supporting ECG changes when available, were included. Exclusion Criteria: (1) History of myocardial infarction, percutaneous coronary intervention (PCI), or coronary artery bypass grafting (CABG). (2) Patients with systemic inflammatory diseases or receiving chronic anti-inflammatory therapy (e.g., corticosteroids, immunosuppressants). (3) Incomplete medical records or non-evaluable CCTA image quality. (4) Anatomical variations of the heart or coronary arteries. (5) Patients who underwent emergency or planned revascularization (PCI or CABG) within 30 days of the index evaluation. (6) MACEs that occurred within the preceding 30 days. A detailed flowchart of the patient selection process and study design is presented in Fig. 3. The diagnosis of unstable angina (UA) was based on the 2021 AHA/ACC/ASE/CHEST/SAEM/SCCT/SCMR guideline for the evaluation and diagnosis of chest pain. UA was defined as new-onset or worsening angina at rest or with minimal exertion, without persistent ST-segment elevation on electrocardiogram and with negative cardiac troponin levels^14^.

### CT scanning protocols

All CCTA scanning was accomplished using dual-source CT of a third‐generation scanner (Somatom Force; Siemens Heathineers, Germany). Prior to the examination, the heart rates of all participants were controlled, and respiratory training was conducted. For patients with a heart rate exceeding 80 beats per minute, metoprolol was administered in doses ranging from 25 to 100 mg, tailored to the individual’s heart rate. Once the heart rate dropped below 80 beats per minute and contraindications for CCTA were ruled out, the patient was positioned supine. The electrode leads were placed on the left anterior chest and connected to the electrocardiogram (ECG) gating system, with the ECG signal adjusted to ensure clear visibility of the R wave for precise scan centering. The scanning protocol utilized prospective ECG gating, with scans performed during end-expiratory breath-holding, achieving a maximum duration of 0.25 s and a single-sector temporal resolution of 66 ms. The tube potential was set within a range of 70–120 kV. The tube current and voltage were adjusted by the operator, considering the patient’s body habitus and clinical requirements, in conjunction with the CT scanner’s automatic exposure control system to optimize the image quality while minimizing the radiation dose. Considering the influence of tube voltage on PCATa, we applied a previously reported correction method, adjusting the average HU values obtained at 70, 80, 90, 100, and 110 kV by dividing by the corresponding correction factors^15^. Diastolic phase images were used for the final CCTA analysis.

### PCAT parameter analyses

**Fig. 4:**
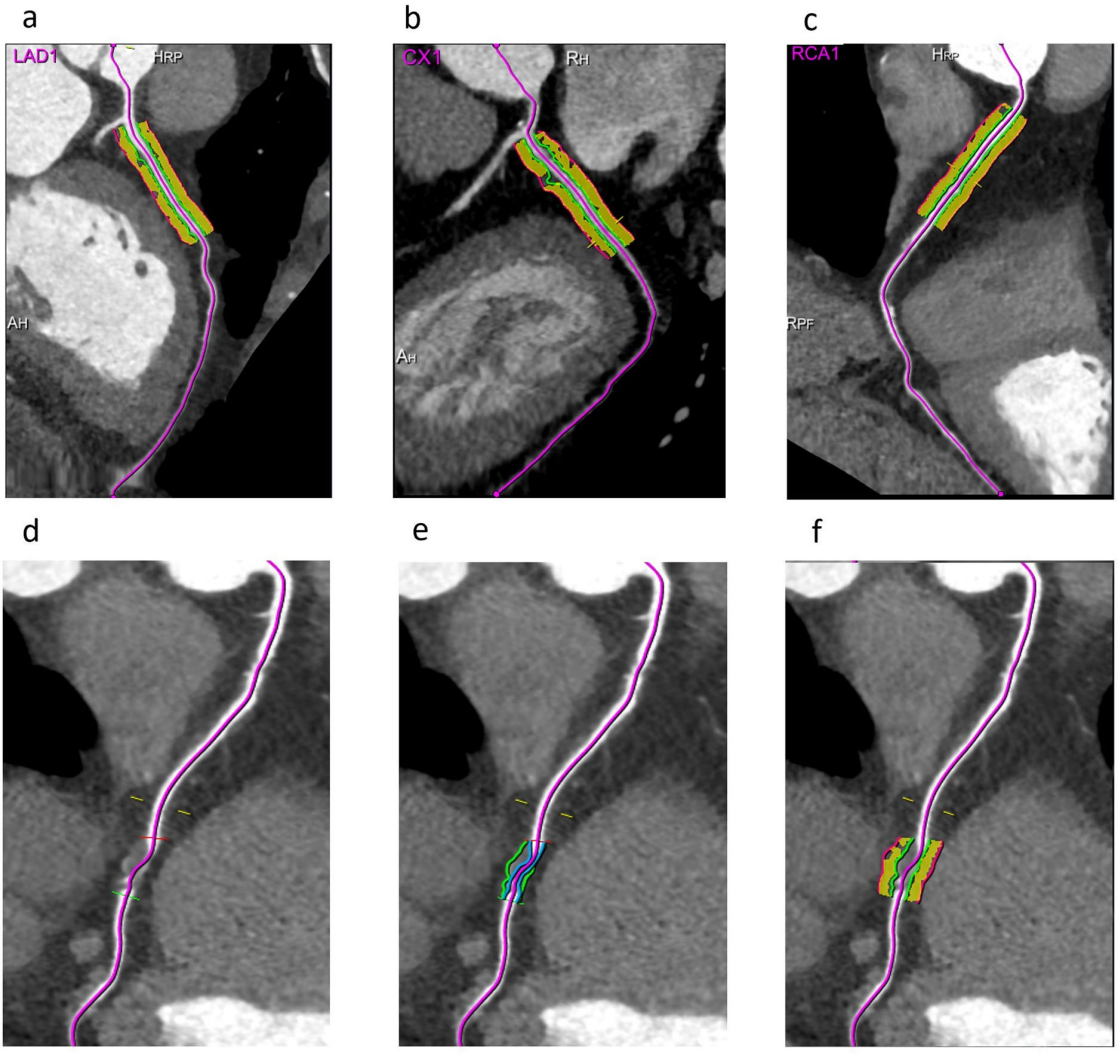
Analysis of proximal 40 mm segments and lesion-specific PCATa. **a**–**c** Schematic diagram of PCATa measurement for the proximal segments of the three coronary arteries (LAD, LCX, RCA) at 40 mm. **d**–**f** Schematic diagram of lesion-specific PCATa measurement. The measurement radial range corresponds to the PCAT within the radial distance of the vessel diameter. *LAD* left anterior descending artery, *LCX* left circumflex artery, *RCA* right coronary artery, *PCATa* pericoronary adipose tissue attenuation.

This study employed dedicated PCATa analysis software (Coronary FAI Analysis, version 1.0.2, Siemens Healthineers) for quantitative assessments. PCATa was defined as the mean CTa of adipose tissue, measured within a radial distance equal to the diameter of the target vessel from the outer vessel wall. The analysis encompassed both the proximal 40 mm of the RCA, LAD, LCX, and lesion-specific segments, focusing on voxels with attenuation values between − 190 and − 30 HU (Fig. 4). Specifically, PCAT was assessed from 10 to 50 mm distal to the ostium for the RCA, and over a 40-mm segment starting at the left main coronary bifurcation for the LAD and LCX. Additionally, the PCAT volume values within the proximal 40-mm segments of the three coronary arteries were also recorded. Lesion-specific PCAT was defined as the average attenuation of the adipose tissue surrounding the parent vessels where the plaques were located^16^, with the proximal and distal boundaries manually delineated. In each patient, lesion-specific PCATa was measured by identifying the segment with the most severe stenosis in each of the three major coronary arteries (LAD, LCX, RCA), if applicable. For patients with plaques in more than one coronary artery, lesion-specific PCATa was calculated as the mean of the PCATa values from all selected segments^16^, thereby providing an integrated estimate of the overall perivascular inflammatory burden. Data measurements were performed manually by two cardiovascular radiologists with 3 and 12 years of experience in cardiac imaging. The two radiologists, who were blinded to the clinical history and clinical outcomes of the patients, independently analyzed the lesions. The average PCATa and volume values measured by both observers were recorded for subsequent analyses. Inter-observer and intra-observer variability of PCAT measurements was assessed by comparing data from two independent observers using 40 randomly selected PCAT measurement values, with blinding and a minimum interval of two weeks. Variability was calculated using the formula: CV (%) = (s/X) × 100, where s is the standard deviation (SD) and X is the mean of the cross-sectional areas; precision was expressed as an average %CV. When the CV was below 15%, the initial measurements were considered as the final PCAT.

### CAD-RADS analyses

According to the CAD-RADS^17^, a software application (Syngo.via, version 21 A, Siemens Healthineers, Germany) was utilized to assess and classify CAD severity based on CCTA. In this study, patients with coronary artery stenosis > 50% were included and CAD-RADS categories 0–2 were excluded. The CAD-RADS categories 4A and 4B were combined into a single group representing severe stenosis^10^. Therefore, the CAD-RADS classifications in our study were divided into three levels: CAD-RADS 3 (50–69% stenosis: moderate stenosis), CAD-RADS 4 (70–99% stenosis: severe stenosis or left main coronary artery stenosis > 50% or three-vessel stenosis ≥ 70%), with CAD-RADS 5 indicating complete occlusion (100% stenosis).

### Follow-up

Follow-up data were obtained from hospital records, clinic visits, and telephone interviews. The follow-up duration was calculated starting from the first examination of CCTA until an endpoint occurred or the last contact with the patient. The primary endpoint of this study was MACE, defined as all-cause mortality, sudden cardiac death, heart failure requiring hospitalization, unplanned coronary revascularization, acute myocardial infarction, or recurrent ventricular arrhythmia. Unplanned coronary revascularization was defined as any revascularization that occurred due to clinical deterioration and excluded procedures which were planned on the basis of the index coronary angiography results.

The secondary endpoints were defined as a composite of all-cause mortality, sudden cardiac death, and acute myocardial infarction, which were considered hard endpoints according to established cardiovascular outcome definitions.

### Statistical analysis

All statistical analyses were conducted using SPSS (Version 26.0, SPSS, Chicago, IL) and R Studio (version 4.3.3). Normally distributed variables were reported as the mean ± SD and compared between groups using independent-sample Student’s t-tests. Non-normally distributed variables were presented as medians and interquartile ranges and compared using the Mann–Whitney U test. Categorical variables were reported as frequencies and percentages and compared using Chi-square tests. The Cox regression analysis was employed to determine the impact of clinical characteristics, CCTA parameters, and PCAT parameters on MACE, with results expressed as HRs and 95% CIs. Multivariate analyses were performed using a forward stepwise selection approach to identify the independent predictors of MACE. Univariate and multivariate analyses were conducted on a per-vessel basis, with each coronary artery (LAD, LCX, and RCA) assessed independently. Kaplan–Meier curves were used to estimate the cumulative incidence of MACE, and log-rank tests were performed for comparisons. The Youden index was utilized to identify the optimal cutoff points. To evaluate the prognostic value of PCATa surrounding the proximal 40-mm segments of the three major coronary arteries and lesion-specific PCAT, different multivariate Cox regression models were constructed: Model 1 employed a forward stepwise selection strategy to identify clinical risk factors; Models 2, 3, 4, and 5 extended Model 1 by incorporating the proximal 40-mm segments of the LAD, LCX, RCA, and lesion-specific PCAT, respectively. The incremental value of each model was assessed by calculating the global Chi-square statistic and improvements in the C-index. *P* < 0.05 was considered statistically significant. The reclassification performance of each model was compared using the integrated discrimination improvement and the category-free net reclassification index.

## Supplementary Information

Below is the link to the electronic supplementary material.

Supplementary Material 1

Supplementary Material 2

## Data Availability

The datasets used and analyzed during the current study are available from the corresponding author on reasonable request.
