# Supplementary material for: Prognostic value of lesion-specific and proximal coronary segment pericoronary adipose tissue CT Attenuation in ischemic heart disease with angina pectoris
Source: Sci Rep. 2025 Nov 24;15:41558. doi: 10.1038/s41598-025-25445-y (PMC12644825; doi:10.1038/s41598-025-25445-y)
Supplement: Supplementary file 1 — Supplementary Material 1 [file 41598_2025_25445_MOESM1_ESM.docx]

| **Supplementary Table S1** Inter- and Intra-rater Reliability of PCAT Measurements at Different Coronary Artery Segments | | | | | | |
| --- | --- | --- | --- | --- | --- | --- |
|  | Inter- rater | | | Intar- rater | | |
|  | CV% | ICC (95%CI) | P value | CV% | ICC (95%CI) | P value |
| LAD proximal 40 mm PCATa | -1.40 | 0.968(0.941~0.983) | < 0.001 | -2.85 | 0.844(0.774~0.931) | < 0.001 |
| LCX proximal 40 mm PCATa | -2.00 | 0.915(0.845~0.954) | < 0.001 | -5.43 | 0.821(0.687~0.901) | < 0.001 |
| RCA proximal 40 mm PCATa | -1.48 | 0.970(0.944~0.984) | < 0.001 | -3.06 | 0.911(0.838~0.952) | < 0.001 |
| Lesion-specific PCATa | -1.32 | 0.960(0.926~0.979) | < 0.001 | -3.29 | 0.856(0.679~0.818) | < 0.001 |
| Notes: LAD, Left Anterior Descending Artery; LCX, Left Circumflex Artery; RCA, Right Coronary Artery; PCATa, Pericoronary Adipose Tissue Attenuation; CV, Coefficient of Variation; ICC, Intraclass Correlation Coefficient; 95%CI, 95% confidence interval. | | | | | | |

| **Supplementary Table S2** Univariable and Multivariable Cox Proportional Hazard Regression Analyses for secondary endpoints | | | | |
| --- | --- | --- | --- | --- |
|  | **Univariable Analysis** | | **Multivariable Analysis** | |
|  | HR (95% CI) | *P* value | HR (95% CI) | *P* value |
| Age (per 1 yr increase) | 1.021 (0.985 ~ 1.058) | 0.262 | - | - |
| Gender(male) | 2.768 (1.096 ~ 6.987) | 0.031 | - | - |
| BMI (per 1 kg/m2) | 0.897 (0.768 ~ 1.049) | 0.173 | - | - |
| Diabetes | 2.359 (1.007 ~ 5.523) | 0.048 | - | - |
| Hypertension | 1.007 (0.425 ~ 2.388) | 0.987 | - | - |
| History of smoking | 3.443 (1.471 ~ 8.058) | 0.004 | - | - |
| History of alcohol consumption | 2.378 (0.978 ~ 5.784) | 0.056 | - | - |
| TG, mmol/L | 1.154 (1.001 ~ 1.330) | 0.049 | - | - |
| Unstable angina | 10.946 (3.249 ~ 36.870) | < 0.001 | 10.872 (3.089 ~ 38.266) | < 0.001 |
| PCATa, HU |  |  |  |  |
| LAD proximal 40 mm | 1.037 (0.981 ~ 1.095) | 0.198 | - | - |
| LCX proximal 40 mm | 1.065 (1.004 ~ 1.129) | 0.036 | - | - |
| RCA proximal 40 mm | 1.082 (1.035 ~ 1.131) | < 0.001 | 1.084 (1.026 ~ 1.146) | 0.004 |
| Lesion-specific | 1.071 (1.021 ~ 1.123) | 0.005 | - | - |
| PCAT volume, mm3 |  |  |  |  |
| LAD proximal 40 mm | 0.998 (0.997 ~ 0.999) | < 0.001 | 0.998 (0.997 ~ 0.999) | 0.004 |
| LCX proximal 40 mm | 0.999 (0.998 ~ 0.999) | 0.040 | - | - |
| RCA proximal 40 mm | 0.999 (0.999 ~ 0.999) | 0.038 | - | - |
| CAD-RADS category |  |  |  |  |
| 3 | 1 (reference) | NA | 1 (reference) | NA |
| 4 | 16.402 (3.799 ~ 70.824) | < 0.001 | 5.961 (1.010 ~ 35.182) | 0.049 |
| 5 | 16.791 (3.062 ~ 92.086) | 0.001 | - | - |
| Notes: BMI, Body Mass Index; TG, Triglycerides; PCATa, Pericoronary Adipose Tissue Attenuation; LAD, Left Anterior Descending Artery; LCX, Left Circumflex Artery; RCA, Right Coronary Artery; CAD-RADS, Coronary Artery Disease Reporting and Data System. | | | | |

| **Supplementary Table S3** Univariable and Multivariable Cox regression analysis of predictors for MACE after censoring early revascularization (within 90 days post-CCTA). | | | | |
| --- | --- | --- | --- | --- |
|  | **Univariable Analysis** | | **Multivariable Analysis** | |
|  | HR (95% CI) | *P* value | HR (95% CI) | *P* value |
| Age (per 1 yr increase) | 1.014 (0.992 ~ 1.037) | 0.223 | - | - |
| Gender(male) | 1.381 (0.823 ~ 2.317) | 0.222 | - | - |
| BMI (per 1 kg/m2) | 0.964 (0.876 ~ 1.061) | 0.457 | - | - |
| Diabetes | 2.200 (1.293 ~ 3.744) | 0.004 | 1.579 (0.899 ~ 2.772) | 0.112 |
| Hypertension | 1.117 (0.645 ~ 1.935) | 0.693 | - | - |
| History of smoking | 1.604 (0.966 ~ 2.665) | 0.068 | - | - |
| History of alcohol consumption | 1.895 (1.050 ~ 3.420) | 0.034 | 1.579 (0.899 ~ 2.772) | 0.122 |
| TG, mmol/L | 1.119 (1.004 ~ 1.247) | 0.042 | 1.086 (0.962 ~ 1.225) | 0.182 |
| Unstable angina | 3.304 (1.913 ~ 5.704) | < 0.001 | 2.890 (1.646 ~ 5.075) | < 0.001 |
| PCATa, HU |  |  |  |  |
| LAD proximal 40 mm | 1.048 (1.014 ~ 1.083) | 0.006 | 1.028 (0.983 ~ 1.076) | 0.221 |
| LCX proximal 40 mm | 1.065 (1.027 ~ 1.105) | < 0.001 | 1.028 (0.984 ~ 1.074) | 0.208 |
| RCA proximal 40 mm | 1.065 (1.035 ~ 1.097) | < 0.001 | 1.078 (1.032 ~ 1.126) | < 0.001 |
| Lesion-specific | 1.066 (1.034 ~ 1.099) | < 0.001 | 1.060 (1.026 ~ 1.095) | < 0.001 |
| PCAT volume, mm3 |  |  |  |  |
| LAD proximal 40 mm | 0.999 (0.998 ~ 0.999) | < 0.001 | 0.999 (0.999 ~ 1.000) | 0.187 |
| LCX proximal 40 mm | 0.999 (0.998 ~ 0.999) | 0.002 | 0.999 (0.999 ~ 1.000) | 0.091 |
| RCA proximal 40 mm | 0.999 (0.999 ~ 0.999) | 0.049 | 1.000 (1.000 ~ 1.001) | 0.433 |
| CAD-RADS category |  |  |  |  |
| 3 | 1 (reference) | NA | 1 (reference) | NA |
| 4 | 3.590 (2.029 ~ 6.350) | < 0.001 | 1.916 (0.889 ~ 4.127) | 0.097 |
| 5 | 3.183 (1.325 ~ 7.643) | 0.010 | 1.202 (0.390 ~ 3.705) | 0.749 |
| Notes: BMI, Body Mass Index; TG, Triglycerides; PCATa, Pericoronary Adipose Tissue Attenuation; LAD, Left Anterior Descending Artery; LCX, Left Circumflex Artery; RCA, Right Coronary Artery; CAD-RADS, Coronary Artery Disease Reporting and Data System. | | | | |

| **Supplementary Table S4** Distribution of Plaque Locations in Three Coronary Arteries | | | |
| --- | --- | --- | --- |
|  | All patients  N=213 | MACE  N=72 | NO MACE  N=141 |
| **Plaque location, n (%)** |  |  |  |
| LAD | 179 | 65 | 114 |
| Proximal segment | 66(36.87) | 26(40.00) | 40(35.09) |
| Middle Segment | 83(46.37) | 27(41.54) | 56(49.12) |
| Distal Segment | 41(22.90) | 16(24.61) | 25(17.73) |
| LCX | 108 | 57 | 51 |
| Proximal segment | 39(36.11) | 18(31.58) | 21(41.18) |
| Middle Segment | 51(47.22) | 24(42.11) | 27(52.94) |
| Distal Segment | 30(27.78) | 19(33.33) | 11(21.57) |
| RCA | 145 | 61 | 84 |
| Proximal segment | 58(40.00) | 27(44.26) | 31(36.90) |
| Middle Segment | 59(40.69) | 20(32.79) | 39(46.43) |
| Distal Segment | 36(24.83) | 19(31.15) | 17(20.24) |
| Notes:LAD, Left Anterior Descending Artery; LCX, Left Circumflex Artery; RCA, Right Coronary Artery. | | | |
