# Supplementary material for: Prognostic value of lesion-specific and proximal coronary segment pericoronary adipose tissue CT Attenuation in ischemic heart disease with angina pectoris
Source: Sci Rep. 2025 Nov 24;15:41558. doi: 10.1038/s41598-025-25445-y (PMC12644825; doi:10.1038/s41598-025-25445-y)
Supplement: Supplementary file 2 — Supplementary Material 2 [file 41598_2025_25445_MOESM2_ESM.docx]

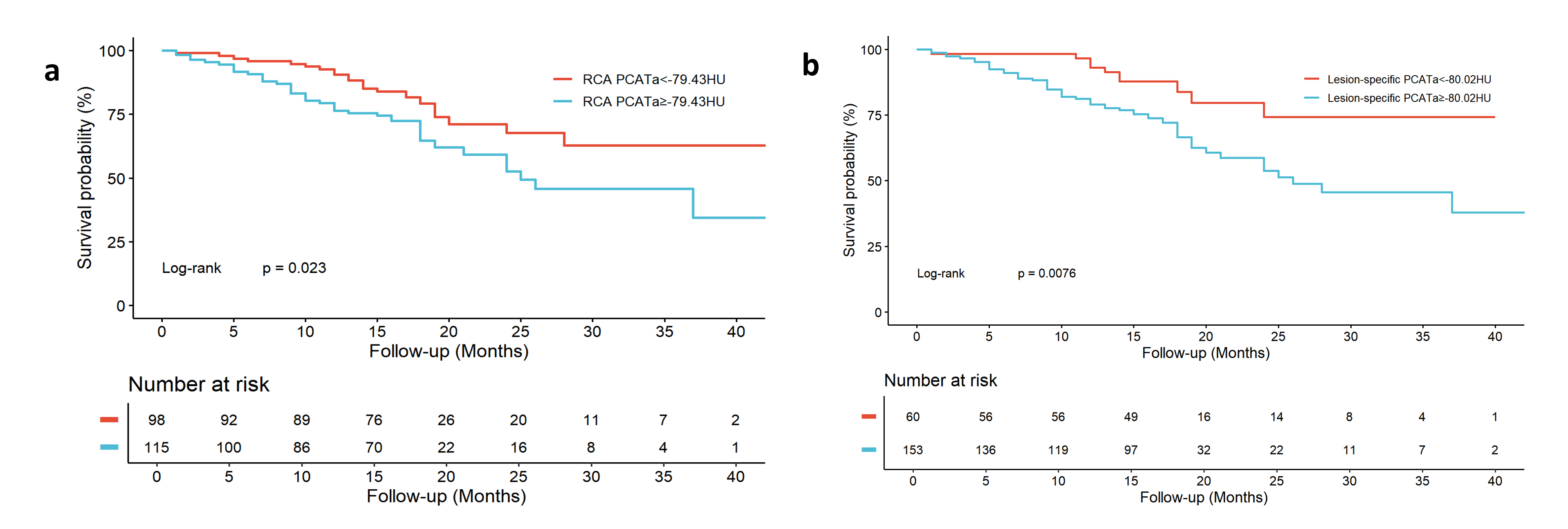


**Supplementary Figure S1** Kaplan–Meier curves illustrating MACE-free survival stratified by PCAT attenuation thresholds **after censoring patients who underwent early coronary revascularization (within 90 days post-CCTA)**. **(a)** Patients with RCA-PCATa > –79.43 HU exhibited significantly lower MACE-free survival compared to those with lower values. **(b)** Lesion-specific PCATa > –80.02 HU was similarly associated with increased MACE risk.

**
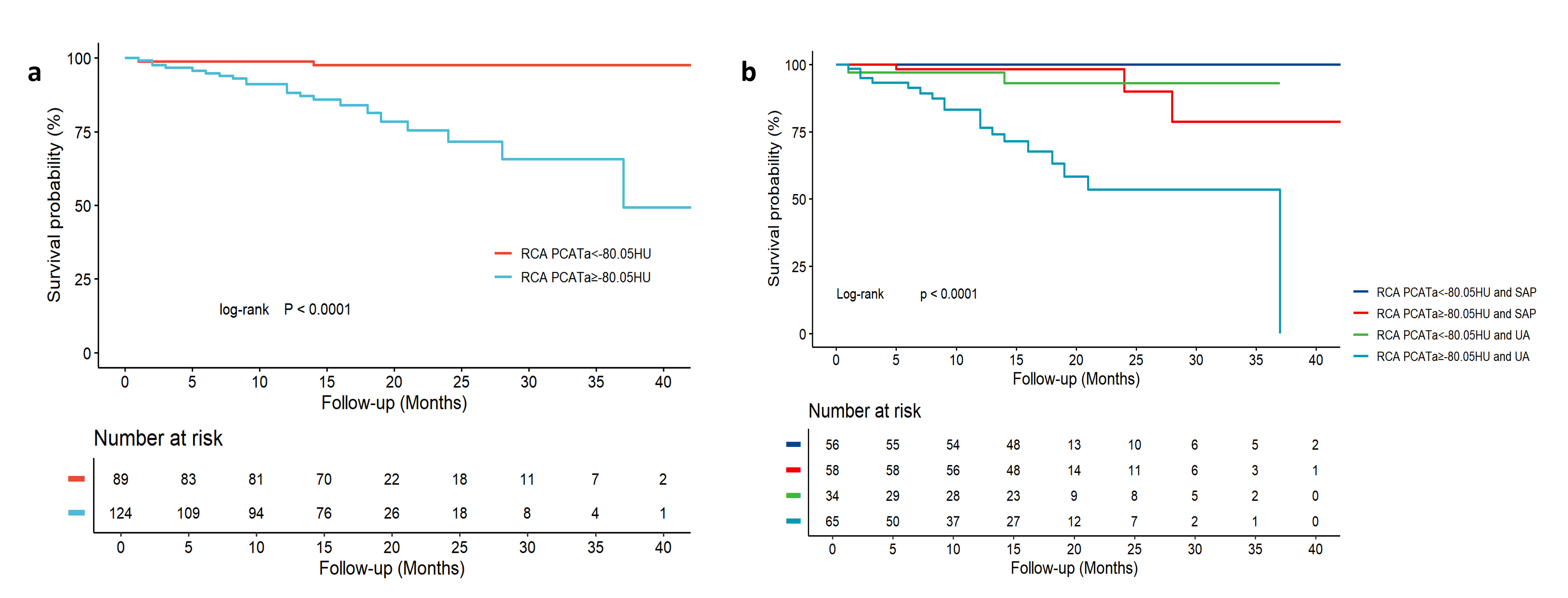
**

**Supplementary Figure S2 (a)** Kaplan-Meier curves showing event-free survival probability for **secondary endpoints**, stratified by RCA-PCATa levels (≥–80.05 HU vs. <–80.05 HU). **(b)** Cumulative survival curves for **secondary endpoints** based on the combined stratification of clinical presentation (stable vs. unstable angina) and RCA-PCATa level. The number of patients at risk is shown below the x-axis at each time point.
